# Diagnostic Accuracy of Multiparametric Ultrasound Versus Cross-Sectional Imaging for Differentiating Benign and Malignant Cervical Lymph Nodes: A Histopathology-Referenced Retrospective Study

**DOI:** 10.3390/diagnostics16193115

**Published:** 2026-09-25

**Authors:** Inva Muçi, Steffen Dommerich, Philipp Arens, Markus Herbert Lerchbaumer, Thomas Fischer, Erin Sprünken, Heidi Olze, Katharina Margherita Wakonig

**Affiliations:** 1 Charité—University Medical Center Berlin, Corporate Member of Freie Universität Berlin and Humboldt-Universität zu Berlin, Department of Otorhinolaryngology, Charitéplatz 1, 10117 Berlin, Germany; 2 Charité—University Medical Center Berlin, Corporate Member of Freie Universität Berlin and Humboldt-Universität zu Berlin, Department of Radiology, Charitéplatz 1, 10117 Berlin, Germany; 3 Charité—University Medical Center Berlin, Corporate Member of Freie Universität Berlin and Humboldt-Universität zu Berlin, Institute of Biometry and Clinical Epidemiology, Charitéplatz 1, 10117 Berlin, Germany

**Keywords:** diagnostic accuracy, multiparametric ultrasound, contrast-enhanced ultrasound, cross-sectional imaging, cervical lymphadenopathy, cervical lymph nodes, histopathology, head and neck cancer

## Abstract

**Background/Objectives**: Accurate differentiation between benign and malignant cervical lymphadenopathy is a central task in head and neck imaging. This distinction is relevant in oncologic follow-up and in the general evaluation of enlarged cervical lymph nodes (CLNs), as nodal status influences prognosis, further diagnostic work-up, and treatment planning, including invasive diagnostic procedures or surgery. Current cross-sectional imaging approaches show variable diagnostic performance. This study aimed to compare the diagnostic accuracy of multiparametric ultrasound (mpUS) and cross-sectional imaging for the differentiation of benign and malignant CLNs. **Methods**: Data from a consecutive series of patients who underwent mpUS examination of a CLN, including B-mode, color-coded duplex sonography (CCDS), shear-wave elastography (SWE) and contrast-enhanced ultrasonography (CEUS) were retrospectively analyzed at the lymph-node level. Patients were eligible if complete mpUS and cross-sectional imaging documentation before tissue sampling was available. Histopathologic reports served as the reference standard. Accuracy, sensitivity, specificity, positive predictive value (PPV), and negative predictive value (NPV) were analyzed. **Results**: A total of 97 CLNs from 92 patients were analyzed; 33 CLNs were benign. Compared with cross-sectional imaging, mpUS showed higher accuracy by 15.5% (95% CI, 6.0–24.9%), higher sensitivity by 12.5% (95% CI, 2.5–23.4%), with higher PPV and NPV. In the exploratory analysis, CEUS and SWE improved the discriminatory power of B-mode and CCDS, with the complete mpUS protocol providing the best overall discrimination. **Conclusions**: MpUS showed higher accuracy, sensitivity, PPV and NPV than cross-sectional imaging. These findings suggest that mpUS may help characterize enlarged CLNs during diagnostic work-up and oncologic follow-up.

## 1. Introduction

Cervical lymph node (CLN) enlargement is a common presenting symptom and often the first clinical sign of a malignant neoplasm in head and neck oncology centers. CLN enlargement has a broad differential diagnosis, including reactive, infectious, granulomatous, lymphoproliferative and metastatic causes [1]. In primary care settings, malignancies account for only 4% of cervical lymphadenopathy (CLA) in patients older than 40 years [2]. In contrast, metastases as well as other malignant causes account for approximately 40% of all CLA presenting in head and neck oncology centers, as reported by multiple studies [1,3,4,5,6].

Within this subgroup, head and neck squamous cell carcinoma (HNSCC) accounts for up to 50% of the malignancies [1,3,4,7]. From an HNSCC-specific perspective, regional nodal metastases are already present at diagnosis in approximately half of cases, as reported in the Surveillance, Epidemiology, and End Results (SEER)-based cohort of 40,164 patients [8]. In addition, approximately 30% of clinically node-negative necks harbor occult metastases [9].

Another study reported that 31% of patients referred for suspected head and neck cancer over a one-year period presented with CLN enlargement as their only symptom [7]. Accurate differentiation between benign and malignant CLNs therefore remains an important and common clinical challenge in the initial diagnostic evaluation, as it directly influences treatment decisions, which may include invasive surgery. CLN assessment is also crucial in oncologic surveillance, where treatment response and subsequent management are evaluated. This is mainly due to the prognostic significance of nodal status, which is a major determinant of overall survival [10,11].

For the nodal staging of oro- and hypopharyngeal carcinoma, the German S3 guideline recommends cross-sectional imaging with computed tomography (CT) or magnetic resonance imaging (MRI) from the skull base to the upper thoracic aperture. However, it also notes that CT, MRI, and ultrasound (US) show comparable diagnostic accuracy in differentiating between benign and malignant CLNs [12]. Similarly, the American Academy of Otolaryngology–Head and Neck Surgery (AAO-HNSF) guideline for adult neck masses recommends contrast-enhanced CT or MRI in patients at increased risk of malignancy but recognizes the role of US for superficial neck pathology and image-guided sampling [13].

By contrast, the UK National Multidisciplinary Head and Neck cancer guideline places US directly within the diagnostic pathway for neck lumps, including suspected nodal metastatic disease. It recommends same-day US and, where indicated, US-guided fine-needle aspiration cytology (FNAC) or core biopsy [14]. This aligns with the National Health Service (NHS) Faster Diagnosis Standard [15], which aims for cancer diagnosis or exclusion within 28 days of referral. Consistent with this pathway rationale, dedicated clinics for the evaluation of CLA report high diagnostic yield of US, streamlined care [7] and a definite diagnosis rate of 93.9% in one study [16].

This highlights the practical advantages of US in the assessment of CLNs: it is time-efficient and enables real-time bedside evaluation by the treating physician or radiologist, thereby addressing the importance of a prompt evaluation of suspected malignancies. In addition, US is widely available, cost-effective, radiation-free [17] and particularly well suited in the evaluation of superficial structures [18]. Another important advantage of US in head and neck settings is that it can be performed directly by the treating surgeon [19].

Three meta-analyses comparing the diagnostic performance of conventional US, CT, MRI and positron emission tomography with or without computed tomography (PET/CT or PET) for differentiating benign from malignant CLNs have reported broadly overlapping results with substantial variability within individual modalities. Across these studies, the sensitivity of conventional US ranged from 66% to 87%, with specificity ranging from 78% to 86%. For CT, MRI and PET or PET/CT, reported sensitivities ranged from 52% to 81%, while specificities ranged from 63% to 93% [20,21,22]. These findings suggest that current imaging approaches remain diagnostically imperfect, leaving room for improvement in the assessment of CLNs in patients with HNSCC as well as in the broader diagnostic work-up for suspicious CLNs.

Multiparametric ultrasound (mpUS) integrates advanced techniques such as shear-wave elastography (SWE) to assess tissue stiffness and contrast-enhanced US (CEUS) to evaluate microvascularization. It is particularly applicable to superficial structures already routinely evaluated with US, including CLNs [23]. Studies have shown a high diagnostic performance of these two techniques for malignancy differentiation across all lymph node locations [24,25] and specifically in the head and neck area, making them a promising addition to conventional US for the evaluation of CLNs. SWE has also been increasingly applied beyond lymph-node imaging, including in musculoskeletal structures and the thyroid gland, highlighting its broad potential for noninvasive tissue characterization [26,27].

SWE has been reported to achieve a sensitivity of 81% and specificity of 85% in diagnosing malignant CLNs [28]. Similarly, a recent meta-analysis reported a sensitivity of 84% and specificity of 87% for CEUS when assessed as a standalone modality [29].

However, the central concept of mpUS is to provide an integrated assessment of multiple distinct lymph-node features, thereby leveraging the diagnostic strengths of the individual ultrasound modalities. Malignant CLNs differ from benign CLNs in several sonographic features, including morphology, vascular architecture, stiffness, and perfusion [30]. B-mode depicts lymph node morphology, color-coded duplex sonography (CCDS) characterizes vascular architecture, SWE evaluates stiffness, and CEUS depicts microperfusion and enhancement patterns within the lymph node [31]. The ability to assess each lymph node feature with the modality particularly suited to visualizing it represents one of the key advantages of mpUS. This was supported by one of our previous studies, in which the mpUS assessment of CLNs showed a sensitivity of 97% [32].

Although both comprehensive mpUS and cross-sectional imaging are used for CLN assessment, comparative evidence based on direct lymph-node-level matching with histopathology remains limited. Therefore, the primary aim of this study was to compare the diagnostic accuracy of mpUS with that of cross-sectional imaging in differentiating malignant from benign CLNs. Secondary endpoints included the comparison of sensitivity, specificity, positive predictive value (PPV) and negative predictive value (NPV) between the two imaging approaches. A further secondary analysis assessed whether the inclusion of more than one lymph node per patient materially influenced the results.

One exploratory analysis compared the diagnostic performance of the individual mpUS modalities with that of cross-sectional imaging across the prespecified diagnostic parameters. Another exploratory post hoc analysis was performed to assess the incremental diagnostic information of SWE and CEUS.

## 2. Materials and Methods

### 2.1. Study Design

This study was designed as a retrospective single-center diagnostic accuracy study based on a previously established prospective patient cohort [32,33], with additional retrospective extraction of radiologic imaging reports. The mpUS imaging records of the CLNs from the prospective cohort were scanned for eligibility and analyzed qualitatively, reflecting routine clinical decision-making. Our unit of analysis was the cervical lymph node. Histopathologic findings served as the reference standard against which mpUS and radiologic findings were compared. This study was reported in accordance with the Standards for Reporting Diagnostic Accuracy Studies (STARD) 2015 guidelines [34].

In the initial prospective study, after recruitment, patients underwent a standardized mpUS examination and marking of the target CLN(s). Histopathological examination served as the reference standard. Tissue was obtained by biopsy, lymph-node excision or neck dissection. If a patient’s health did not allow for surgery, biopsies were obtained by US-guided core needle biopsies.

The current study was approved by the institutional ethics committee of Charité–Universitätsmedizin Berlin (protocol code: 2026-173-CCM, date of approval: 7 July 2026).

### 2.2. Patient Cohort

In the initial prospective study, a consecutive series of patients was recruited by the outpatient clinic of the Otorhinolaryngology Department at Charité–Universitätsmedizin Berlin (Campus Charité Mitte) from July 2019 to December 2021. Potentially eligible individuals were identified from patients presenting to the clinic.

The following inclusion criteria were applied: (i) age 18 or older, (ii) either enlarged CLN(s) for more than 2 weeks or suspicious CLN(s) during oncologic follow-up and (iii) consent to histological confirmation through biopsy, lymph-node excision, neck dissection or US-guided core needle biopsy. In the initial prospective study, 105 patients were enrolled.

For the present retrospective analysis, 99 patients with 104 CLNs were available for analysis. Patients were included if they met the following criteria: (i) complete mpUS documentation for the CLN before tissue sampling and (ii) corresponding radiologic assessment of the cross-sectional examination before tissue sampling.

### 2.3. Data Collection

For our retrospective analysis, the institutional imaging database Picture Archiving and Communication System (PACS) was searched to retrieve the mpUS records for each CLN. The extracted images relied on the protocol followed in the prospective study, as reported by Wakonig et al. [32]. Briefly summarized, all mpUS examinations were performed before histological confirmation using the same device, probe, and setup (4–10 MHz multifrequency linear array transducer with a center frequency of 7 MHz, Acuson Sequoia, Siemens Healthineers, Erlangen, Germany). MpUS was performed according to a standard protocol by an ear, nose and throat (ENT) specialist (European Federation of Societies for Ultrasound in Medicine and Biology [EFSUMB] Head and Neck Level II) or a radiologist (EFSUMB Level II), both with extensive experience in neck US. The mpUS protocol included B-mode US, CCDS, SWE, and CEUS. Figure 1 depicts an example of the mpUS documentation for one benign and one malignant CLN.

From each CLN examination, B-mode images of the target CLN in two planes with and (where available) without measurements were retrieved and analyzed.

CCDS was used to determine the vascularization of the CLN. Doppler signal assessment was performed using the available archived imaging material, including video clips and, where applicable, static images documenting the CCDS signal.

SWE was performed with minimal pressure using the two-dimensional (2D) SWE approach, obtaining five consecutive images. Circular regions of interest (ROIs) were placed within the lymph node and adapted to its size for the quantitative analysis performed in one of our previous studies [33]. The US device measures shear-wave propagation velocities and displays them as color-coded elastography maps, corresponding to the elastic properties of the tissue [35]. The ultrasound system encoded different levels of tissue stiffness using the following colors:

• Blue: soft tissue, area with low resistance, indicating high elasticity.
• Green: moderately soft tissue, area with slightly increased resistance, showing reduced elasticity.
• Yellow: hard tissue, area with significantly reduced elasticity, indicating greater stiffness.
• Red: very hard tissue, area with minimal elasticity or maximum resistance, indicating extreme stiffness.

The SWE map is then placed in dual-image mode alongside the corresponding B-mode image, with ROIs placed on both images, as shown in Figure 1e,f. Three of the five acquired images were analyzed for the qualitative analysis performed in the present study. The ROIs placed within each CLN were only used to assess the quantitative SWE measurements presented in [33]; these results are not part of the present study.

The same high-end system was used for CEUS examinations. A state-of-the-art CEUS-specific protocol and a low mechanical index (<0.1) were used to avoid early microbubble destruction. A 90 s (s) cine loop was recorded while a bolus of 1.2 mL of a second-generation ultrasound contrast agent (SonoVue^®^, Bracco Imaging, Milan, Italy) was injected intravenously followed by a 10 mL flush of 0.9% saline solution. The contrast agent consists of microbubbles filled with sulfur hexafluoride and is eliminated through the lungs within 10–15 min (min). For the present analysis, the CEUS recording of the target CLN was analyzed.

The radiologic assessments of CT, MRI, PET/CT or positron-emission tomography/magnetic resonance imaging (PET/MRI) examinations performed prior to tissue sampling were retrieved from the institution’s patient record system. Imaging acquisition characteristics were extracted from the available examination documentations. In the case of several available cross-sectional examinations, the examination closest to the tissue sampling date was taken. Cross-sectional imaging was evaluated by (i) radiologists in training (years 1–5), with subsequent review by consultant radiologists with more than 10 years of clinical experience, or (ii) board-certified radiologists. For the dichotomous comparison with mpUS, radiologic reports were classified as either radiologically suspicious (malignant) or non-suspicious for malignant involvement (benign). A report was classified as indicating malignancy if (i) the corresponding CLN was explicitly described as malignant, or suspicious for malignant involvement, or (ii) histopathologic examination of the corresponding CLN was recommended because malignant involvement could not be excluded.

A report was classified as radiologically non-suspicious (or benign) if it (i) explicitly described the target CLN as benign, (ii) stated that there was no evidence of cervical lymph-node metastasis or (iii) provided only descriptive information, such as CLN size, morphology, or anatomical location, without expressing concern for malignancy.

The histopathologic reports were also retrieved from the institution’s patient record system. Histopathologic findings were classified as malignant when the report documented malignant involvement of the lymph node, either as metastatic disease from any primary tumor or as lymphoma. Histopathology was used as the reference standard because it allows direct microscopic confirmation or exclusion of malignant involvement in the examined CLN. Adverse events related to the index tests or the histopathologic reference standard were not systematically assessed in this retrospective diagnostic accuracy study.

Target lymph nodes were matched across mpUS, CT/MRI/PET, and histopathology by laterality and Robbins neck level. No patient had more than one included target node within the same side-specific neck level.

### 2.4. Reader Workflow

Imaging records of each CLN from all ultrasound modalities (B-mode, CCDS, SWE, CEUS) were reviewed independently by an experienced ENT specialist (EFSUMB Head and Neck Level II) and a novice reader (medical student with limited prior US experience). For each US modality, imaging findings were classified as either benign or suspicious for malignancy according to the criteria described in Section 2.5. Imaging interpretation. Subsequently, a final mpUS classification was assigned to the CLN. This was based on an integrated assessment of all available ultrasound features across the four modalities and was not determined by any single modality or by a predefined combination rule.

After the initial independent assessments, all image sets were jointly re-evaluated by the two readers, and consensus classifications were established for each modality as well as for the overall mpUS classification. The same predefined criteria and result classification as those used for the individual assessments were applied.

During mpUS review, both readers were blinded to the patient history, histopathologic diagnosis, and to the corresponding cross-sectional imaging reports. The experienced reader had performed part of the original ultrasound examinations approximately five years before the retrospective review. Given this long interval and the blinded reassessment based on predefined criteria, the risk of recall bias was considered low, although it cannot be completely excluded. Histopathologic assessment served as the reference standard and was performed by the institution’s Department of Pathology as part of routine diagnostic work-up with access to relevant clinical information.

### 2.5. Imaging Interpretation

Each image or video sequence was analyzed according to the criteria described below. No formal scoring system or decision rule was applied. Instead, each modality was assessed using an integrated qualitative interpretation of predefined sonographic features (see Table 1).

For B-mode ultrasound, the evaluated features included CLN shape, size, margins, presence of a fatty hilum and internal echotexture. A round lymph node configuration was generally considered suspicious for malignancy [30,36,37,38]. The cutoff value for the short-axis diameter (SAD) was set at 10 mm, in accordance with the guidance from the German Cancer Society (Deutsche Krebsgesellschaft, DKG) [39]. This threshold was chosen as reported values in the literature range from approximately 5 to 13 mm, without a universally accepted SAD cutoff [30,37].

A 15 mm long-axis diameter (LAD) cutoff was also evaluated [40]. However, long-axis diameter alone has limited discriminatory value, as CLN size varies with anatomical location and benign reactive enlargement may exceed conventional thresholds, whereas metastatic nodes may remain below them. Therefore, LAD was interpreted in conjunction with morphologic features such as shape, border characteristics, internal heterogeneity/necrosis, and other suspicious imaging findings rather than as a stand-alone criterion. Irregular margins were considered suspicious for malignancy [28,34,35]. Ill-defined or blurred margins were also assessed as features potentially supporting malignancy. Nonetheless, they were interpreted with caution because while they may suggest extracapsular spread, they also occur in benign or reactive CLNs [37,38]. The presence of a fatty hilum was interpreted as a feature favoring benign morphology [30]. Regarding echotexture, a heterogeneous appearance was considered suspicious for malignancy [36].

CCDS findings were classified as hilar or nonhilar/absent flow. Purely hilar flow was regarded as a benign vascular pattern. Absent and nonhilar vascular patterns, including peripheral and mixed hilar–peripheral flow, were considered suspicious for malignancy [36,41].

SWE was evaluated qualitatively based on the color map. In our ultrasound system, blue with light-green areas corresponded to softer tissue and was therefore interpreted as more consistent with benign CLN morphology. Conversely, focal or diffuse areas coded as red, yellow, or dark green corresponded to increased tissue stiffness and were interpreted as features supporting malignancy [30,42]. However, qualitative SWE assessment of lymph nodes remains challenging because there is no universally accepted standard across ultrasound systems. Consequently, neither the qualitative interpretation of the color map nor the quantitative interpretation of absolute cutoff values is directly interchangeable between ultrasound systems, as both color encoding and measured stiffness values may vary between devices. SWE findings were therefore interpreted with caution, and borderline stiffness patterns were considered less diagnostically decisive than clearly increased nodal stiffness.

In the CEUS recordings, the perfusion appearance of each CLN was analyzed. A centripetal perfusion pattern (enhancement from the periphery toward the center) and heterogeneous contrast distribution were interpreted as suspicious for malignant involvement. Perfusion defects were defined as focal non-enhancing or avascular areas within the CLN. These areas were interpreted as suggestive of necrosis and considered suspicious for malignancy, particularly when present together with other suspicious sonographic features [29,43].

None of the above-mentioned features was used as a standalone criterion. Instead, they served as a guide for the structured assessment of each ultrasound modality. The final classification of each CLN was based on the overall sonographic appearance across all evaluated features, both for the individual modalities and for mpUS. This integrated approach was intended to reflect a real-world diagnostic workflow. Nevertheless, standardized criteria are needed in future studies to improve reproducibility and comparability. An example of the mpUS imaging records of a benign and a malignant CLN is shown in Figure 1.

### 2.6. Statistical Analysis

The sample size was determined by the number of available CLNs that fulfilled the eligibility criteria for the retrospective analysis. Cross-sectional imaging findings that did not unequivocally fulfill criteria for malignancy were classified according to the predefined binary decision rules. Therefore, no separate indeterminate category was used for the index tests. No reference standard data were indeterminate or missing for the included CLNs. In cases of missing mpUS or cross-sectional imaging data, the corresponding CLNs were excluded from the analysis.

For the primary analysis, each CLN was classified as benign or malignant by mpUS and cross-sectional imaging and compared with the histopathologic reference standard. The mpUS examinations, which had been performed as additional study examinations, were re-evaluated by two readers in consensus using the defined criteria also based on our previous mpUS studies of CLNs [32,33]. Cross-sectional imaging was not retrospectively re-interpreted for this study. Instead, the corresponding official radiology reports available in PACS were used, as these had been generated during clinical routine according to established radiologic lymph node criteria and formally approved by a board-certified radiologist. Accuracy, sensitivity, specificity, PPV, and NPV were calculated for each modality using contingency tables. For the individual diagnostic performance parameters, 95% confidence intervals (CIs) were calculated using the Wilson score method for binomial proportions.

Differences in diagnostic performance parameters between mpUS and cross-sectional imaging were calculated as mpUS minus cross-sectional imaging. Because both modalities were evaluated on the same CLNs, paired methods were used for all comparative analyses. For accuracy, sensitivity and specificity, 95% CIs for the paired differences in proportions were reported with 95% confidence intervals using Newcombe’s method 10 for paired binomial proportions [44]. The corresponding *p*-values were obtained using two-sided exact McNemar tests.

For PPV and NPV, 95% CIs for the differences were calculated using paired bootstrap resampling. The corresponding *p*-values were obtained using two-sided paired Monte Carlo permutation tests with 10,000 random permutations. A two-sided significance level of α = 0.05 was used to define statistical significance in this study.

Inter-reader agreement before consensus was assessed using Cohen’s kappa. Diagnostic performance of each reader, percentage agreement and the number of discrepant assessments were also calculated.

To assess whether the inclusion of more than one CLN per patient materially influenced the results, a sensitivity analysis was conducted after excluding all patients who contributed more than one lymph node. The complete statistical analysis pathway described above was repeated using this restricted dataset.

In an additional exploratory analysis, the diagnostic performance of B-mode US, CCDS, SWE and CEUS as standalone modalities was calculated and compared with that of cross-sectional imaging using the same statistical analysis pathway.

In the exploratory post hoc analysis, the apparent discrimination of logistic regression models was evaluated using the area under the receiver operating characteristic curve (ROC AUC), with 95% CIs derived from 5000 whole-patient bootstrap resamples. Incremental contributions of SWE and CEUS were assessed using Firth penalized likelihood-ratio tests, and the AUCs of the SWE- and CEUS-augmented models were compared using a two-sided centered patient-bootstrap test.

Contingency tables were plotted using IBM SPSS Statistics, Version 31.0.0.0 (IBM Corp., Armonk, NY, United States of America [USA]). All other statistical analyses were performed with R, Version 4.6.0 (R Foundation for Statistical Computing, Vienna, Austria), using RStudio, Version 2026.05.0+218 (Posit Software, PBC, Boston, MA, USA).

## 3. Results

### 3.1. Study Population and Data Characteristics

From the initial prospective study, ultrasound findings of 104 CLNs from 99 patients were scanned for eligibility. After applying the eligibility criteria, the current study cohort comprised 97 CLNs from 92 patients. A total of five patients contributed two CLNs to the dataset. Three patients each contributed two distinct target nodes from different neck levels on the same examination. The two remaining patients each contributed two CLNs from two distinct examinations, 18 months and 8 months apart, respectively. The mpUS reviewers were blinded to the relation between these nodes.

The flow of participant inclusion and exclusion is presented in Figure 2.

The mean patient age was 59.3 years (standard deviation [SD], 16.6). There were 32 female and 60 male patients.

The corresponding cross-sectional examinations comprised 51 CT, 38 MRI, 7 PET/CT, and one PET/MRI. Table 2 shows a summary of the cross-sectional imaging method distribution and the total number of false CLN classifications for each. Imaging acquisition characteristics are depicted in Appendix A, Table A9.

As tissue sampling, two patients underwent US-guided core needle biopsy (14-gauge needle; Magnum™ Reusable Core Biopsy Instrument, minimum of three specimens) and 90 patients received a neck dissection. The histopathologic characteristics of the examined CLNs are shown in Table 3. Only characteristics that were retrospectively available in the electronic patient record system were extracted.

### 3.2. Diagnostic Parameters for mpUS and Cross-Sectional Imaging

MpUS showed good diagnostic performance with an accuracy of 86.6% (95% CI, 78.4–92.0%), a sensitivity of 96.9% (95% CI, 89.3–99.1%) and a specificity of 66.7% (95% CI, 49.6–80.2%). PPV was 84.9% (95% CI, 75.0–91.4%) and NPV was 91.7% (95% CI, 74.2–97.7%).

Cross-sectional imaging of the same nodes showed an accuracy of 71.1% (95% CI, 61.4–79.2%), a sensitivity of 84.4% (95% CI, 73.6–91.3%) and a specificity of 45.5% (95% CI, 29.8–62.0%). PPV was 75% (95% CI, 63.9–83.6%) and NPV was 60% (95% CI, 40.7–76.6%).

The 2 × 2 contingency tables underlying all diagnostic accuracy estimates are provided in Appendix A, Table A1 and Table A2.

Before consensus, the readers agreed on the overall mpUS classification in 73/97 examinations (75%; κ = 0.19, 95% CI, −0.10–0.47). Agreement across the individual ultrasound modalities ranged from 62% to 75% (κ range, 0.08–0.26), with agreement for qualitative SWE interpretation of 62% (κ = 0.24, 95% CI, 0.05–0.43). Reader-specific diagnostic performance and complete modality-specific agreement results, including the number of discrepant assessments, are presented in Appendix A, Table A7 and Table A8.

### 3.3. Difference in Diagnostic Performance of mpUS and Cross-Sectional Imaging

Accuracy was 15.5% higher for mpUS compared with cross-sectional imaging (95% CI, 6.0–24.9%; *p* = 0.002). Similarly, sensitivity was higher by 12.5% (95% CI, 2.5–23.4%; *p* = 0.021). Specificity also was 21.2% higher for mpUS (95% CI, −0.1–39.8%; *p* = 0.092). Figure 3 depicts an example of an mpUS examination and the corresponding cross-sectional imaging of a malignant CLN.

PPV was 9.9% higher in mpUS than in cross-sectional imaging (95% CI, 2.1–18.3%; *p* = 0.016). Likewise, NPV was 31.7% higher for mpUS (95% CI, 12.5–52.0%; *p* = 0.002). These results are compiled in Table 4.

The median interval between mpUS and tissue sampling was 1 day (interquartile range [IQR], 1–2 days; range, 0–20 days). The mean interval was 1.6 days (standard deviation [SD], 2.2 days).

The median interval between cross-sectional imaging examinations and tissue sampling was 23 days (IQR, 15–34 days; range, 1–218 days). The mean interval was 32.3 days (SD, 37 days).

### 3.4. Analysis Restricted to Patients Contributing a Single Lymph Node

For this analysis, the five patients who each contributed two lymph nodes were excluded. This resulted in a restricted dataset of 87 CLNs from 87 patients. The analyses of accuracy, sensitivity, specificity, PPV and NPV were then repeated. The results are presented in Table 5. The 2 × 2 contingency tables underlying the diagnostic accuracy estimates of this sensitivity analysis are provided in Appendix A, Table A3 and Table A4.

### 3.5. Comparison of Individual mpUS Modalities to Cross-Sectional Imaging

The diagnostic performance of each mpUS modality is presented in Appendix A, Table A5. Comparisons between the individual mpUS modalities and cross-sectional imaging are presented in Appendix A, Table A6.

CEUS performed significantly better than cross-sectional imaging in terms of accuracy, sensitivity, PPV and NPV. CEUS showed a 15.5% higher accuracy than cross-sectional imaging (95% CI, 6.4–24.6%; *p* = 0.001) and a 12.5% higher sensitivity (95% CI, 3.9–22.9%; *p* = 0.008). CEUS also had a 9.9% higher PPV (95% CI, 2.2–18.2%; *p* = 0.015) and a 31.7% higher NPV (95% CI, 14.9–50.0%; *p* < 0.001).

Compared with cross-sectional imaging, SWE had a 33.3% higher specificity (95% CI, 13.1–49.8%; *p* = 0.003) and a 12.3% higher PPV (95% CI, 3.8–21.4%; *p* = 0.008).

B-mode US showed a 25% higher NPV than cross-sectional imaging (95% CI, 3.9–46.9%; *p* = 0.036).

### 3.6. Incremental Diagnostic Value of SWE and CEUS

The ROC curves from the exploratory post hoc logistic regression analysis are shown in Figure 4. The reference model, comprising B-mode US and CCDS, yielded an AUC of 0.78 (95% CI 0.68–0.87). The addition of SWE increased the AUC to 0.86 (ΔAUC 0.09, 95% CI 0.03–0.16; *p* < 0.001), whereas the addition of CEUS increased the AUC to 0.84 (ΔAUC 0.06, 95% CI 0.01–0.14; *p* < 0.001). The numerical increase in AUC was higher for SWE by 0.03 (95% CI, −0.04–0.1; *p* = 0.4). The model incorporating all four modalities yielded the highest apparent AUC of 0.89, with the highest increase from the reference model (ΔAUC 0.11, 95% CI 0.04–0.2; *p* < 0.001). Its AUC was also higher than that of the CEUS-only model (ΔAUC 0.07, 95% CI 0.02–0.15; *p* = 0.03) and that of the SWE-only model (ΔAUC 0.12, 95% CI 0.06–0.2; *p* < 0.001).

## 4. Discussion

The results of this study show a higher diagnostic accuracy of mpUS compared with cross-sectional imaging for differentiating benign from malignant CLNs. In addition, mpUS showed better performance across several diagnostic parameters, including sensitivity, PPV and NPV.

The 12.5% higher sensitivity of mpUS suggests that mpUS may be particularly useful in reducing the number of malignant CLNs missed by routine cross-sectional imaging. The higher specificity by 21.2% suggests that mpUS may also reduce the number of benign CLNs incorrectly classified as malignant. However, the difference in specificity did not reach statistical significance (*p* = 0.092) and should therefore be interpreted cautiously. The higher PPV and NPV further indicate that, within this study cohort, mpUS provided a more reliable classification of both positive and negative findings than routine cross-sectional imaging. As PPV and NPV are highly dependent on disease prevalence, these estimates are specific to the selected referral population and should be interpreted primarily in the context of specialized head and neck oncology settings.

Meanwhile, cross-sectional imaging showed a sensitivity of 84.4%, which was within the range reported in previous meta-analyses, whereas specificity was lower at 45.5%. This discrepancy may partly reflect differences in case mix, diagnostic thresholds, and the level of analysis, as the present study included several malignant histologies and evaluated specifically matched target lymph nodes. Additionally, specificity was estimated from only 33 benign lymph nodes, which was reflected in its wide confidence interval. These differences limit direct comparison with pooled meta-analytic performance estimates.

CT and MRI incorrectly classified CLNs in 29% of the respective examinations, while false radiologic reports were less frequent for PET/CT at 14% and also occurred in the single PET/MRI examination. These findings are relevant because cross-sectional imaging remains a central component of guideline-based evaluation of CLNs in patients with head and neck cancer, despite known limitations in lymph node characterization.

MpUS combines morphological, vascular, stiffness- and perfusion-related information, which may partly explain the higher observed diagnostic performance. This is supported by Cai et al. [45], who showed that a diagnostic model based on different US modalities achieved high diagnostic performance in patients with CLA. While previous studies have compared conventional US with CT, MRI, or PET/CT for detecting cervical lymph node metastases in HNSCC, direct comparisons of comprehensive mpUS protocols with cross-sectional imaging remain scarce. Most available mpUS studies focus on the diagnostic value of individual US parameters or compare mpUS with histopathology alone. Thus, the present study addresses an important gap by directly comparing mpUS with corresponding cross-sectional imaging assessments for the same histopathologically verified CLNs.

In a previous study, we reported an accuracy of 83% and a sensitivity of 97% for the complete mpUS examination [32]. Another histopathology-referenced study evaluating CLNs with B-mode ultrasound, SWE and CEUS reported an accuracy of 86% and a sensitivity of 90% for the combined model [46]. These findings are consistent with the present results and support the concept that combining US modalities can improve CLN characterization. Other studies comparing conventional US with cross-sectional imaging modalities have also reported higher diagnostic accuracy of US examinations at the lymph-node level [47,48].

In the assessment of the diagnostic performance of each modality, CEUS showed a higher accuracy, sensitivity, PPV and NPV in comparison with cross-sectional imaging. Hence, perfusion-related information from CEUS examinations may have contributed substantially to the overall performance of mpUS. This is plausible because CEUS allows real-time assessment of lymph-node vascularization, enhancement heterogeneity and non-enhancing areas, which may reflect necrosis or altered tumor perfusion [29,49].

SWE showed higher specificity and PPV. Stiffness-related information may be particularly useful for confirming malignancy rather than excluding it. This is in line with previous studies reporting high specificity and PPV of SWE for malignant superficial lymph nodes [50].

CEUS alone and the full mpUS assessment yielded identical diagnostic performance measures. However, their case-level classifications differed in four lymph nodes, with reciprocal changes that offset each other. In a further evaluation of the additional diagnostic value of mpUS over a standalone CEUS examination, mpUS showed a higher AUC by 0.07, suggesting a slightly greater discriminatory ability for differentiating benign from malignant CLNs despite identical diagnostic performance measures.

Nevertheless, these observations should be interpreted with caution, as the comparisons were exploratory. Although each mpUS modality was assigned a benign or malignant classification according to modality-specific criteria (Table 1), they were reviewed as part of the same lymph node examination. Consequently, the readers’ assessment of each modality was not blinded to findings from the others.

The analysis of incremental discriminatory value yielded the largest increase with the full mpUS model. A clear improvement was also observed when either SWE or CEUS was added to the conventional US model. Although the addition of SWE produced a numerically greater increase than CEUS, the difference between the two modalities was not statistically significant (the confidence interval included 0; *p* = 0.4). Therefore, adding SWE or CEUS either individually or together benefits the model substantially, but these data cannot establish whether SWE or CEUS contribute more in the model’s discriminatory power.

The present findings support the clinical rationale for direct sonographic assessment of CLNs relevant to treatment planning, particularly in specialized head and neck oncology settings. The study population had a relatively high prevalence of malignancy (64/97 CLNs), which may not reflect the case mix of an unselected population presenting with CLA. Because PPV and NPV strongly depend on disease prevalence, the values observed in this study should be interpreted within the context of a specialized referral setting and may not be directly generalizable to broader diagnostic pathways. Within such comparable specialized settings, previous studies have reported time-saving effects and improvements in patient care after the integration of surgeon-performed US into head and neck cancer clinics [51,52].

Nonetheless, differentiating benign from malignant cervical lymph nodes is clinically relevant in several settings of head and neck practice. In patients presenting with cervical lymphadenopathy, assessment of nodal morphology helps guide the subsequent diagnostic work-up. In patients with a known malignancy in the head and neck area, CLN evaluation is essential for staging and treatment planning, particularly by identifying lymph nodes suspicious for metastatic involvement. This distinction is clinically important, as nodal metastasis may alter therapeutic decision-making and indicate invasive procedures such as neck dissection.

Morphologic CLN evaluation is also important in oncologic aftercare, where it contributes to the detection of residual or recurrent disease and supports decisions regarding further diagnostic or therapeutic management [53]. Several guidelines recommend regular specialist follow-up visits every 2–3 months during the first two years after treatment, together with one cross-sectional imaging examination within 3–6 months after therapy completion [12,14,54].

However, recurrence is not limited to this early post-treatment period. More than half of recurrences occur beyond 6 months and local or regional recurrence accounts for a substantial proportion of them [55,56]. This highlights the importance of accessible and individualized diagnostic tools in surveillance. In this context, the recommendations from the same guidelines recognize the use of ultrasound in oncologic follow-up and emphasize practical advantages such as availability, cost-effectiveness, safety, and accuracy.

Compared with the literature and in line with previous studies [57,58], our findings indicate a better diagnostic performance of mpUS than conventional US. As outlined in the introduction, conventional US has already been reported to achieve diagnostic performance comparable to cross-sectional imaging in CLN assessment. However, the present results do not imply that mpUS should replace cross-sectional imaging, which remains necessary for evaluating deep anatomical structures and overall disease extent. Rather, they support mpUS as a reliable complementary diagnostic tool for the assessment of CLNs.

This diagnostic accuracy study was a retrospective single-center study conducted in a specialized head and neck clinic, which limits the generalizability of its findings. The specialized clinical setting may also have contributed to selection bias, as patients referred to this clinic are more likely to present with suspicious or complex findings requiring specialized diagnostic evaluation or treatment. Therefore, the study population may not fully represent an unselected population of patients with CLA. Nevertheless, the findings remain clinically relevant, as mpUS is most likely to be used in specialized centers involved in the oncologic management of head and neck diseases and in the diagnostic evaluation of patients with CLN enlargement. Furthermore, the device-dependent nature of SWE color encoding and stiffness measurements may limit the generalizability of these findings to other ultrasound systems.

Another factor that may limit the validity of the findings is the inherent operator dependence of US image acquisition and interpretation [59]. To maximize internal validity, all mpUS examinations included in this study were performed by one of two highly experienced examiners according to a predefined protocol, as described in Section 2.3. The images were subsequently assessed by an experienced reader and a medical student, who served as a structured second reviewer providing an independent assessment and helping to identify ambiguous findings that required discussion. All diagnostic performance measures were calculated from the final consensus image interpretation rather than from the individual reader assessments.

The κ values for pre-consensus agreement indicated limited agreement beyond chance. The qualitative SWE assessment showed the lowest observed percentage agreement (62%; κ = 0.24), supporting the need for standardized interpretation criteria and reader training. However, these findings apply only to this experienced–novice reader pair and cannot be generalized to agreement between experienced ultrasound readers. Furthermore, because the primary diagnostic-performance estimates were based on consensus classifications, they may differ from performance achieved by readers working independently.

The limited sample size may also have affected the precision of the findings. This was particularly relevant for specificity, as the low number of benign lymph nodes resulted in a wider confidence interval and may have influenced the non-significant difference in specificity. Another methodological consideration was the inclusion of five patients who each contributed two lymph nodes, resulting in a lack of complete statistical independence between observations. To evaluate whether this affected the results, a sensitivity analysis was conducted after excluding these patients, as shown in Appendix A, Table A3 and Table A4. This analysis showed only minor differences across diagnostic parameters, with changes of approximately 2–3% for most parameters. These small changes suggest that the inclusion of patients contributing more than one lymph node did not materially alter the overall findings.

Additionally, the cross-sectional imaging comparator was heterogeneous, consisting mainly of CT and MRI, with only a small number of PET-based examinations. As these modalities differ in diagnostic performance and are selected according to clinical indication, the pooled comparison should be interpreted as a comparison with routine cross-sectional imaging rather than with one specific modality.

Beyond the modality-related heterogeneity, previous studies have demonstrated that reinterpretation of head and neck CT and MRI examinations by subspecialized radiologists can lead to relevant changes in staging and treatment recommendations [60]. Thus, the use of official clinical reports instead of standardized expert re-readings represents an important limitation of the present comparison, and may have underestimated the diagnostic performance achievable with optimized cross-sectional imaging reassessment. The observed difference may therefore reflect not only the differences in modalities, but also differences in interpretative setting and degree of standardization. Nonetheless, the released cross-sectional imaging reports represent official clinical reports used either for TNM staging in suspected tumor metastases or for presurgical guidance and, as such, must be regarded as valid diagnostic references.

As reported in Section 3.3, the interval between mpUS and tissue sampling was shorter than the interval between cross-sectional imaging and tissue sampling. While mpUS was performed a median of 1 day before histopathology, the corresponding interval for cross-sectional imaging was 23 days and reached 218 days in one case. This difference reflects the design of the initial prospective studies [32,33], in which all patients underwent preoperative mpUS. Meanwhile, cross-sectional imaging had often been performed earlier as part of the routine diagnostic work-up, in some cases prompted by abnormal US findings.

This further highlights the aforementioned limitation of the retrospective study design. During longer intervals, changes in lymph node size and morphology, including disease progression, may have resulted in discrepancies between imaging findings and the subsequent histopathological status. This timing difference may have disadvantaged cross-sectional imaging relative to mpUS and limits the extent to which differences in diagnostic performance can be attributed solely to the imaging modality.

The present results should therefore be interpreted as demonstrating higher diagnostic performance of dedicated mpUS assessment compared with routine clinical cross-sectional imaging reports under the conditions of this study, rather than suggesting superiority of mpUS over temporally matched, standardized cross-sectional imaging assessment. Future prospective studies should address this limitation by using standardized and comparable intervals between each imaging modality and histopathologic assessment.

This timing difference reflects a separate practical aspect of clinical routine: cross-sectional imaging is not always available on demand on the day of treatment planning or surgery. In addition, CT and PET/CT involve ionizing radiation, and therefore require appropriate justification. MRI and PET/MRI are more expensive and time-consuming examinations and may be less well tolerated by some patients. In contrast, mpUS can be performed in outpatient settings and preoperatively as a bedside procedure, allowing real-time evaluation of the target lymph node and potential integration into specialized head and neck oncology workflows. Although head and neck US is part of established radiologic practice, it can also be reliably performed by appropriately trained head and neck physicians. This is reflected in the AAO-HNSF position statement which supports surgeon-performed neck ultrasound [61].

Future multicenter studies are warranted to validate these findings and establish standardized mpUS examination protocols and diagnostic criteria. Moreover, future studies should clarify existing barriers within diagnostic pathways for CLN assessment and explore how these may be overcome.

## 5. Conclusions

This study compared the diagnostic performance of mpUS and cross-sectional imaging for differentiating malignant from benign changes in 97 cervical lymph nodes from 92 patients, using histopathology as the reference standard. It demonstrated the higher accuracy, sensitivity, PPV, and NPV of mpUS. Exploratory analyses suggested that CEUS and SWE may have contributed to the observed performance. Although these findings indicate that mpUS may have potential value in the evaluation of cervical lymph node enlargement and oncologic follow-up, its integration into routine diagnostic pathways should follow external validation in larger multicenter cohorts. To our knowledge, direct histopathology-referenced comparisons of comprehensive mpUS and cross-sectional imaging at the level of corresponding cervical lymph nodes have not previously been reported, making this the first study to address this question.

## Figures and Tables

**Figure 1:**
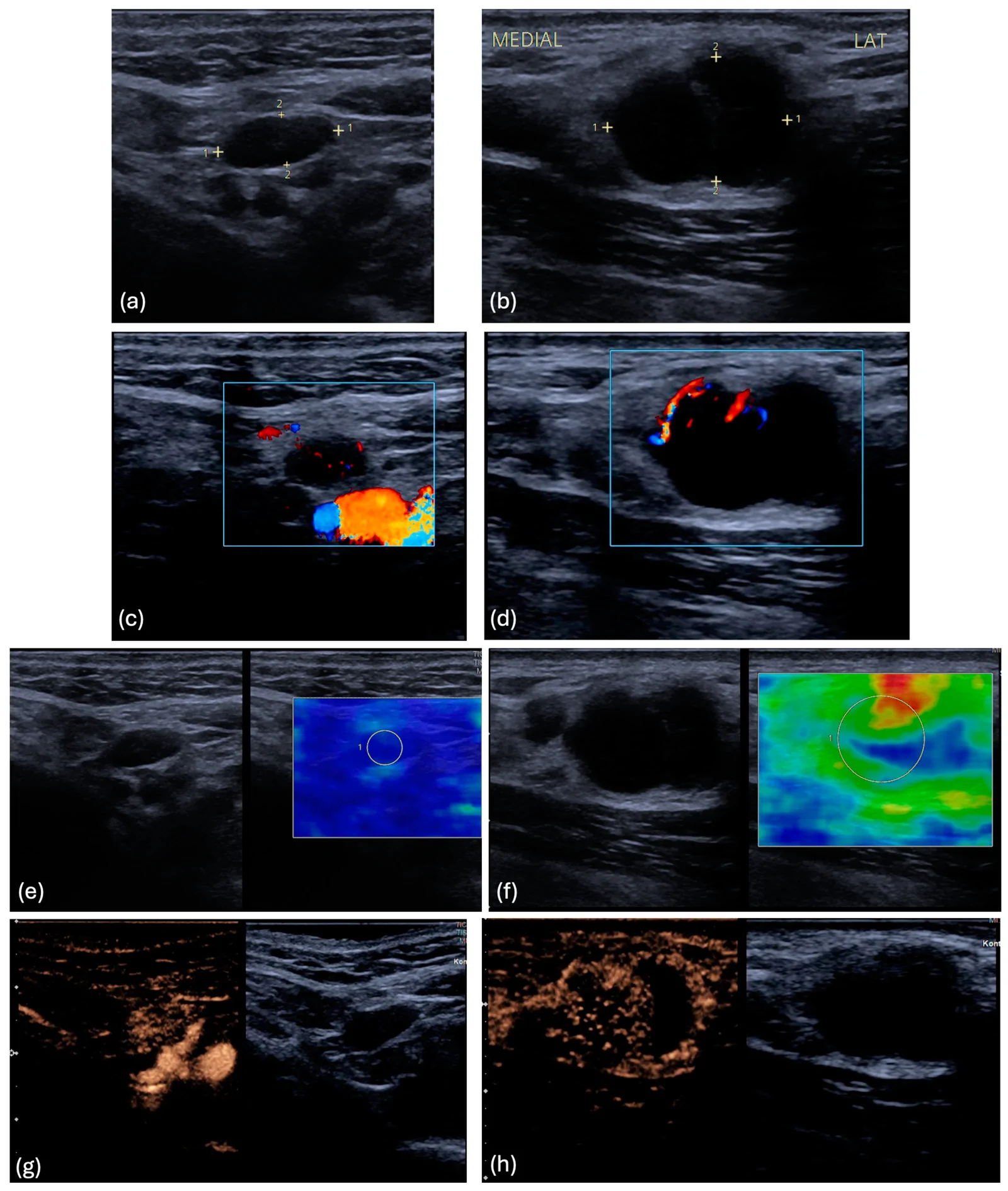
Representative example of multiparametric ultrasound (mpUS) examinations used for evaluation. On the left side a benign cervical lymph node (CLN) is depicted; on the right side a malignant CLN is shown. (**a**,**b**) B-mode ultrasound (US) of the two CLNs. Notice the regular margins in (**a**) and the irregular and ill-defined margins in (**b**). (**c**,**d**) Static images of the color-coded Doppler US (CCDS) depicting vascularization. Notice the full Doppler coverage of the CLN in (**c**) and the mainly peripheral flow in (**d**). (**e**,**f**) Shear-wave elastography (SWE) mapping in dual-image mode with B-mode on the left side. A region of interest (ROI) is set inside the CLN. Notice the blue signal in the CLN in (**e**) and the green and red signal from the CLN in (**f**). (**g**,**h**) Static images of the contrast-enhanced US (CEUS) videos capturing the moment of contrast inflow respectively at 21 and 23 seconds (s), respectively, of the 90 s cine loop. Notice the homogeneous contrast coverage of the CLN in (**g**) and the avascular area on the right half of the CLN in (**h**).

**Figure 2:**
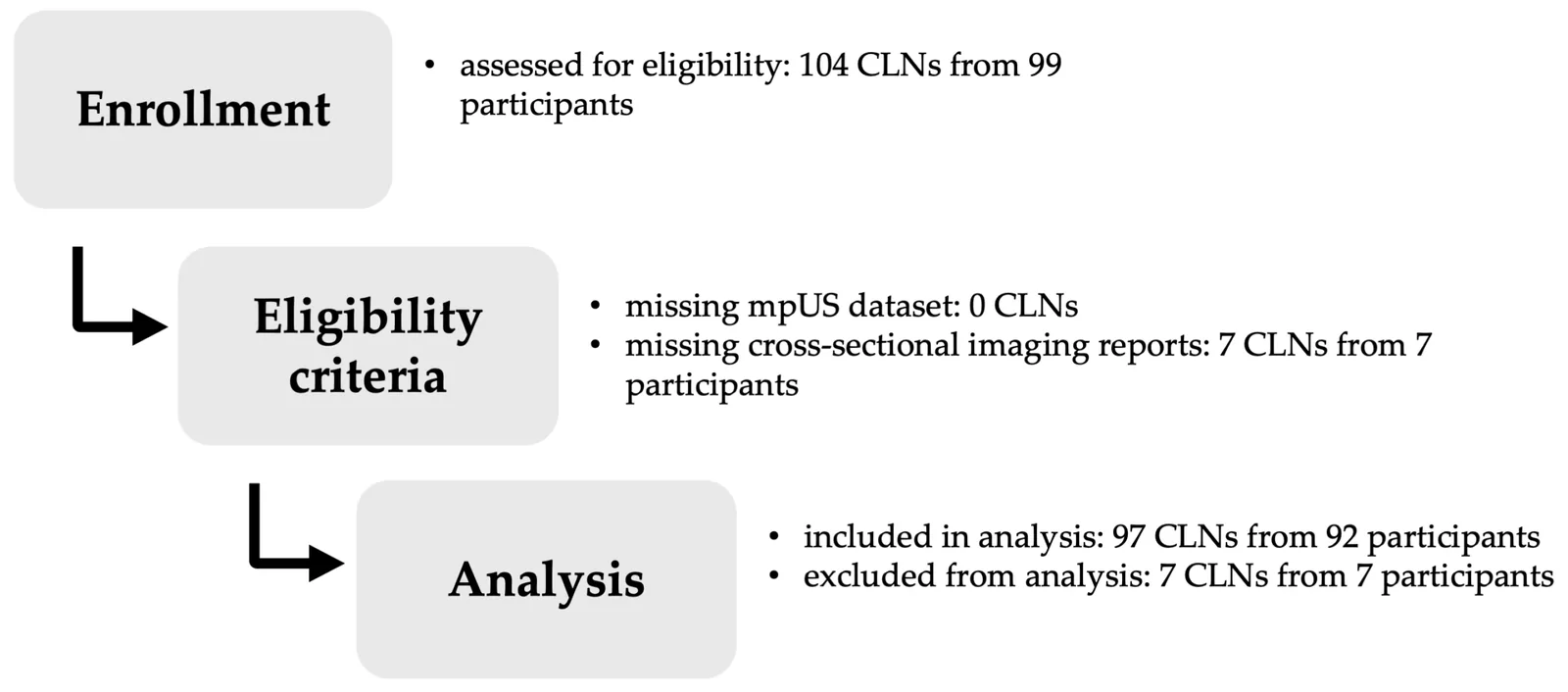
Flow of participants.

**Figure 3:**
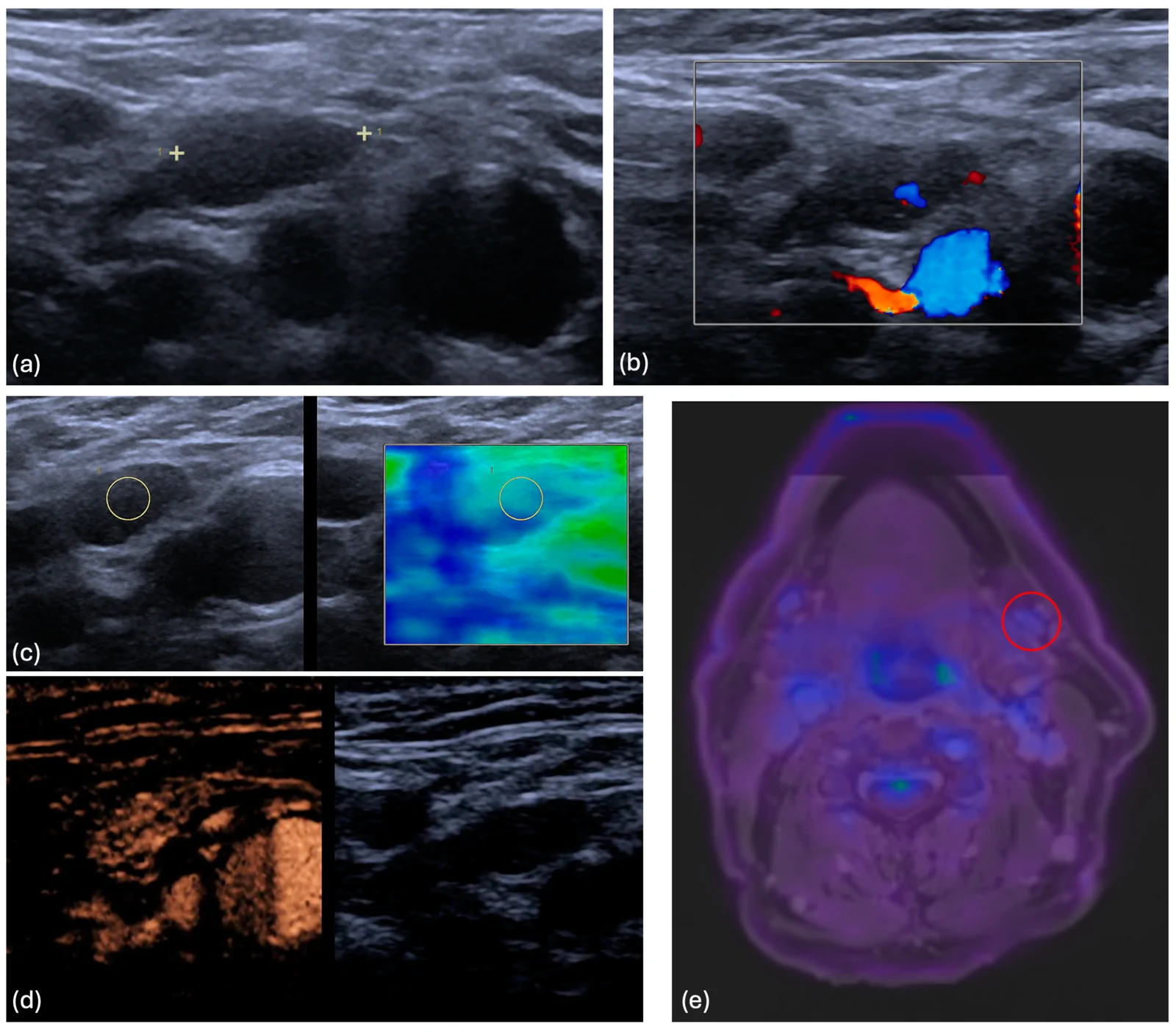
Metastatic CLN from hypopharyngeal squamous cell carcinoma (HNSCC) in neck level II on the left side, depicted by mpUS and cross-sectional imaging. (**a**) B-mode US of the lymph node. Notice the irregular and ill-defined borders. (**b**) Static CCDS image demonstrating blood flow originating from two distinct regions within the CLN. (**c**) SWE mapping demonstrates a predominantly green signal within the CLN. The yellow circle represents the ROI. Measurements obtained from this ROI were not analyzed in the present study. (**d**) Static CEUS image acquired at 20 s demonstrating a small non-enhancing area along the deep border of the CLN. (**e**) Corresponding PET/MRI image of the CLN, with the target CLN encircled in red. While the CLN was suspicious on mpUS, the radiology report stated, “No evidence of lymph nodes suspicious for metastasis.” Histopathologic workup confirmed a metastatic lymph node from the known hypopharyngeal HNSCC.

**Figure 4:**
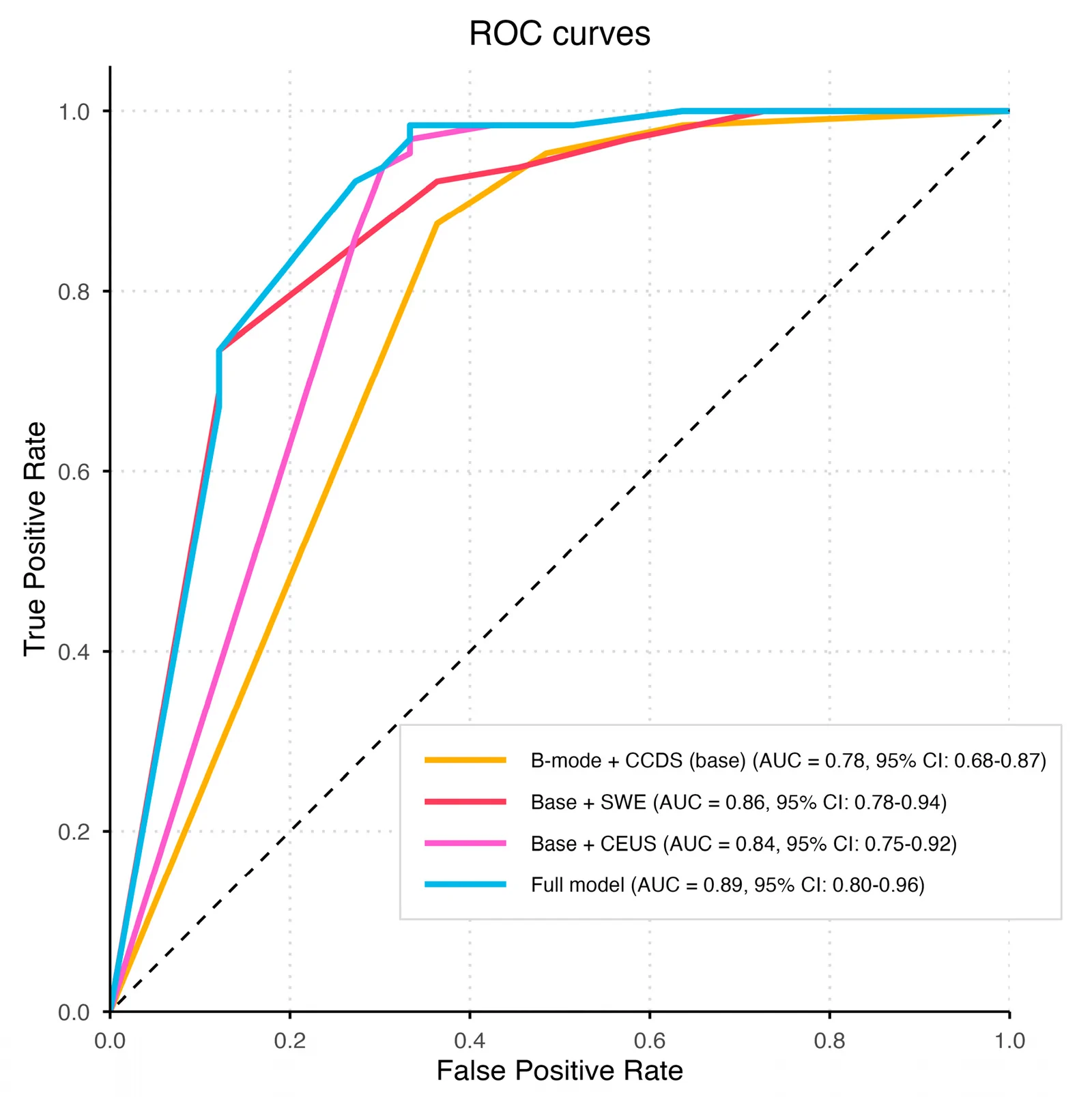
Receiver operating characteristic (ROC) curves assessing the incremental discriminatory value of SWE and CEUS for differentiating benign from malignant CLNs. The reference (“base”) model comprised conventional B-mode ultrasonography and CCDS. Areas under the ROC curve (AUCs) with 95% confidence intervals are reported in the legend. The dashed diagonal line represents chance-level discrimination.

**Table 1:** Rating criteria used for interpretation of mpUS datasets.

US Modality	Criterion	Benign	Malignant
B-mode	Shape	Oval	Round
	Size	SAD ≤ 10 mm, LAD ≤ 15 mm	SAD > 10 mm, LAD > 15 mm
	Margins	Regular, well-defined	Irregular, ill-defined
	Fatty hilum	Present	Absent
	Echotexture	Homogeneous	Heterogeneous
CCDS	Vascularity	Hilar flow	Absent or nonhilar flow
SWE	Tissue stiffness	Soft (blue, light green)	Stiff (dark green, yellow, red)
CEUS	Pattern	Centrifugal	Centripetal
	Distribution	Homogeneous	Heterogeneous
	Defects	Absent	Avascular areas

Abbreviations: SAD = short-axis diameter, LAD = long-axis diameter.

**Table 2:** Distribution of the cross-sectional imaging modalities in this study and the proportion of false reports from each modality.

Cross-Sectional Imaging Modality	Total Examinations/Incorrect Radiologic Reports (%)
CT	51/15 (29%)
MRI	38/11 (29%)
PET/CT	7/1 (14%)
PET/MRI	1/1 (100%)

Abbreviations: CT = computed tomography, MRI = magnetic resonance imaging, PET/CT = positron-emission tomography/computed tomography, PET/MRI = positron-emission tomography/magnetic resonance imaging.

**Table 3:** Histopathologic characteristics and severity distribution of the reported lymph nodes.

Histopathologic Results	*n* (%)
Benign lymph nodes	33 (34.0)
SAD > 10 mm	27
Physiological lymph nodes	18
Reactive lymph node changes ^1^	9
Sinus histiocytosis	5
Tuberculous lymphadenitis	1
Malignant lymph nodes	64 (66.0)
Metastases from HNSCC *	28
SAD > 10 mm	17
Metastasis size > 10 mm	12
Extracapsular growth	10
Necrosis	6
Conglomerates	4
Contralateral to the primary tumor site	1
Lymphoma	18
SAD > 10 mm	13
Metastatic melanoma	8
SAD > 10 mm	7
Metastasis size > 10 mm	5
Metastatic pulmonary adenocarcinoma	2
SAD > 10 mm	0
Extracapsular spread	1
Metastatic salivary duct carcinoma	2
SAD > 10 mm	0
Metastasis size > 10 mm	1
Other metastatic carcinomas ^2^	6
SAD > 10 mm	5
Metastasis size > 10 mm	3
Total	97 (100.0)

* Abbreviations: HNSCC = head and neck squamous cell carcinoma. ^1^ Reactive lymph node changes included expanded germinal centers, follicular or interfollicular hyperplasia and unspecific activation. ^2^ One case each originating from the submandibular gland (large-cell eosinophilic carcinoma), breast, kidney (renal cell carcinoma), urothelium, prostate, and skin (cutaneous squamous cell carcinoma).

**Table 4:** Diagnostic performance results of the multiparametric ultrasound (mpUS) and the cross-sectional imaging (expressed in percentages and 95% confidence intervals [CI]), their difference (calculated as mpUS minus cross-sectional imaging, expressed in % and 95% CI), and the *p*-value of the difference.

	mpUS	Cross-Sectional Imaging	Difference	*p*-Value
Accuracy	86.6 (78.4–92.0)	71.1 (61.4–79.2)	15.5 (6.0–24.9)	0.002
Sensitivity	96.9 (89.3–99.1)	84.4 (73.6–91.3)	12.5 (2.5–23.4)	0.021
Specificity	66.7 (49.6–80.2)	45.5 (29.8–62.0)	21.2 (−0.1–39.8)	0.092
PPV	84.9 (75.0–91.4)	75.0 (63.9–83.6)	9.9 (2.1–18.3)	0.016
NPV	91.7 (74.2–97.7)	60.0 (40.7–76.6)	31.7 (12.5–52.0)	0.002

**Table 5:** Diagnostic performance in patients contributing a single lymph node. Results for mpUS and the cross-sectional imaging are expressed in percentages with 95% CI in parentheses. The difference is calculated as mpUS minus cross-sectional imaging and expressed in % with 95% CI in parentheses.

	mpUS	Cross-Sectional Imaging	Difference	*p*-Value
Accuracy	87.4 (78.8–92.8)	73.6 (63.4–81.7)	13.8 (4.3–23.4)	0.008
Sensitivity	98.3 (90.9–99.7)	84.5 (73.1–91.6)	13.8 (4.2–25.1)	0.008
Specificity	65.5 (47.3–80.1)	51.7 (34.4–68.6)	13.8 (−7.8–33.5)	0.344
PPV	85.1 (74.7–91.7)	77.7 (66.1–86.3)	7.3 (−0.6–15.4)	0.079
NPV	95.0 (76.4–99.1)	62.5 (42.7–78.8)	32.5 (14.8–51.9)	0.001

## Data Availability

The data presented in this study are available on request from the corresponding author due to patient privacy.

## Abbreviations

The following abbreviations are used in this manuscript:

AAO-HNSF	American Academy of Otolaryngology–Head and Neck Surgery
AUC	Area under the curve
CCDS	Color-coded duplex sonography
CEUS	Contrast-enhanced ultrasonography
CI	Confidence interval
CLA	Cervical lymphadenopathy
CLN	Cervical lymph node
CT	Computed tomography
DKG	Deutsche Krebsgesellschaft
DWI	Diffusion-weighted imaging
EFSUMB	European Federation of Societies for Ultrasound in Medicine and Biology
ENT	Ear, nose, and throat
^18^F-FDG	^18^F-fluorodeoxyglucose
FNAC	Fine-needle aspiration cytology
HNSCC	Head and neck squamous cell carcinoma
IQR	Interquartile range
LAD	Long-axis diameter
min	Minutes
mpUS	Multiparametric ultrasound
MRI	Magnetic resonance imaging
NHS	National Health Service
NPV	Negative predictive value
PACS	Picture Archiving and Communication System
PET	Positron emission tomography
PET/CT	Positron emission tomography/computed tomography
PET/MRI	Positron emission tomography/magnetic resonance imaging
PPV	Positive predictive value
ROC	Receiver operating characteristic
ROI	Region of interest
S	Seconds
SAD	Short-axis diameter
SD	Standard deviation
SEER	Surveillance, Epidemiology, and End Results
STARD	Standards for Reporting Diagnostic Accuracy Studies
SWE	Shear-wave elastography
T	tesla
US	Ultrasound
USA	United States of America

## Appendix A

**Table A1:** Cross-tabulation of mpUS findings against the histopathologic reference standard.

	Histopathology		
mpUS	Malignant	Benign	Total
malignant	62	11	73
benign	2	22	24
Total	64	33	97

**Table A2:** Cross-tabulation of cross-sectional imaging findings against the histopathologic reference standard.

	Histopathology		
Cross-Sectional Imaging	Malignant	Benign	Total
malignant	54	18	72
benign	10	15	25
Total	64	33	97

**Table A3:** Cross-tabulation of mpUS findings against the histopathologic reference standard for the sensitivity analysis.

	Histopathology		
mpUS	Malignant	Benign	Total
malignant	57	10	67
benign	1	19	20
Total	58	29	87

**Table A4:** Cross-tabulation of cross-sectional imaging findings against the histopathologic reference standard for the sensitivity analysis.

	Histopathology		
Cross-Sectional Imaging	Malignant	Benign	Total
malignant	49	14	63
benign	9	15	24
Total	58	29	87

**Table A5:** Diagnostic performance of all mpUS modalities. Results are expressed in percentages with 95% CI in parentheses.

	B-Mode	CCDS	SWE	CEUS
Accuracy	80.4 (71.4–87.1)	76.3 (66.9–83.6)	76.3 (66.9–83.6)	86.6 (78.4–92.0)
Sensitivity	95.3 (87.1–98.4)	90.6 (81.0–95.6)	75.0 (63.2–84.0)	96.9 (89.3–99.1)
Specificity	51.5 (35.2–67.5)	48.5 (32.5–64.8)	78.8 (62.2–89.3)	66.7 (49.6–80.3)
PPV	79.2 (68.9–86.8)	77.3 (66.7–85.3)	87.3 (76.0–93.7)	84.9 (75.0–91.4)
NPV	85.0 (64.0–94.8)	72.7 (51.8–86.8)	61.9 (46.8–75.0)	91.7 (74.2–97.7)

**Table A6:** Differences in diagnostic performance between individual ultrasound modalities and cross-sectional imaging. Differences were calculated as the diagnostic-performance estimate for each individual ultrasound modality minus the corresponding estimate for cross-sectional imaging. Results are reported as differences in % with 95% confidence intervals and *p*-values in parentheses. Positive values indicate better diagnostic performance of the ultrasound modality, whereas negative values indicate better diagnostic performance of cross-sectional imaging.

	B-Mode	CCDS	SWE	CEUS
Accuracy difference	9.3 (−0.9–19.3; *p* = 0.108)	5.2 (−3.9–14.1; *p* = 0.359)	5.2 (−5.9–16.0; *p* = 0.458)	15.5 (6.4–24.6; *p* = 0.001)
Sensitivity difference	10.9 (0.2–22.2; *p* = 0.065)	6.3 (−4.2–17.1; *p* = 0.344)	−9.4 (−21.8–3.2; *p* = 0.210)	12.5 (3.9–22.9; *p* = 0.008)
Specificity difference	6.1 (−16.1–27.3; *p* = 0.791)	3.0 (−14.9–20.6; *p* = 1.0)	33.3 (13.1–49.8; *p* = 0.003)	21.2 (−0.1–39.8; *p* = 0.092)
PPV difference	4.2 (−3.7–12.3; *p* = 0.312)	2.3 (−4.1–8.9; *p* = 0.543)	12.3 (3.8–21.4; *p* = 0.008)	9.9 (2.2–18.2; *p* = 0.015)
NPV difference	25.0 (3.9–46.9; *p* = 0.036)	12.7 (−6.9–32.8; *p* = 0.263)	1.9 (−15.5–19.4; *p* = 0.830)	31.7 (14.9–50.0; *p* < 0.001)

**Table A7:** Pre-consensus inter-reader agreement for the individual ultrasound modalities and overall mpUS assessment. All 97 CLNs had complete paired assessments from both readers.

Modality	Agreement, *n* (%)	Disagreement, *n* (%)	Cohen’s κ (95% CI)
B-Mode	69 (71%)	28 (29%)	0.08 (−0.21–0.37)
CCDS	62 (64%)	35 (36%)	0.26 (0.07–0.46)
SWE	60 (62%)	37 (38%)	0.24 (0.05–0.43)
CEUS	67 (69%)	30 (31%)	0.21 (−0.02–0.48)
mpUS	73 (75%)	24 (25%)	0.19 (−0.10–0.47)

**Table A8:** Reader-specific diagnostic performance of the pre-consensus overall mpUS classification. Reader 1 was an experienced ENT consultant, and Reader 2 was a medical student with limited prior ultrasound experience. Values are percentages with 95% confidence intervals.

	Reader 1	Reader 2
Accuracy	83.5 (74.9–89.6)	67.0 (57.1–75.6)
Sensitivity	96.9 (89.3–99.1)	89.1 (79.1–94.6)
Specificity	57.6 (40.1–72.8)	24.2 (12.9–41.0)
PPV	81.6 (71.4–88.7)	69.5 (58.9–78.4)
NPV	90.5 (71.1–97.3)	53.3 (30.1–75.2)

**Table A9:** Imaging acquisition characteristics from the radiological reports.

Imaging Acquisition Characteristics	*n* (%)
**CT**	**51 (52.6)**
Intravenous iodinated contrast	
Administered	46
Not administered	1
Not reported	4
Anatomical coverage	
Neck only	13
Extended beyond the neck	35
Not reported	3
**PET/CT**	**7 (7.2)**
Intravenous iodinated contrast	
Administered	5
Not administered	2
Anatomical coverage	
Extended beyond the neck	7
Tracer	
^18^F-FDG *	7
**MRI**	**38 (39.2)**
Field strength	
1.5 T *	14
3 T	7
Not reported	17
Anatomical coverage	
Neck	28
Extended beyond the neck	7
Not reported	3
T1- and T2-weighted sequences	
Both performed	30
Not reported	8
DWI *	
Performed	13
Not performed	15
Not reported	10
Intravenous gadolinium-based contrast	
Administered	27
Not administered	3
Not reported	8
Fat-supressed sequences	
Performed	30
Not reported	8
**PET/MRI**	**1 (1.0)**
Field strength	3 T
Anatomical coverage	Full body
T1- and T2-weighted sequences	Performed
DWI	Performed
Intravenous gadolinium contrast	Administered
Fat supressed sequences	Performed
Tracer	18F-FDG
Total	97 (100.0)

* Abbreviations: T = tesla, ^18^F-FDG = ^18^F-fluorodeoxyglucose, DWI = Diffusion-weighted imaging.

## References

[1] Laffers W., Eggert K., Schildhaus H.U., Bootz F., Gerstner A.O.H. (2012). Histologic diagnoses in persistently swollen cervical lymph nodes. Head Neck.

[2] Fijten G.H., Blijham G.H. (1988). Unexplained lymphadenopathy in family practice. An evaluation of the probability of malignant causes and the effectiveness of physicians’ workup. J. Fam. Pract..

[3] Al Kadah B., Popov H.H., Schick B., Knöbber D. (2015). Cervical lymphadenopathy: Study of 251 patients. Eur. Arch. Otorhinolaryngol..

[4] Bassiouni M., Kang G., Olze H., Dommerich S., Arens P. (2023). The Diagnostic Yield of Excisional Biopsy in Cervical Lymphadenopathy: A Retrospective Analysis of 158 Biopsies in Adults. Ear Nose Throat J..

[5] Celik S., Slipcevic B., Ceviken E., Kılıc O., Kalcioglu M.T. (2025). Cervical lymphadenopathies: A Retrospective Single-Center Analysis of Patients Undergoing Excisional Biopsy. Med. Bull. Sisli Etfal Hosp..

[6] Preethi S., Narayanan N., Venkatakarthikeyan C. (2024). A Prospective Clinicopathological Study of Cervical Lymphadenopathy in a Head and Neck Unit of a Tertiary Referral Centre. Apollo Med..

[7] Chew A., Costa I., Sen I., Dewage A.E., Ibrahim A., Metcalfe C., McMorran J.P., George A. (2026). Service Evaluation of a “Straight to Scan” Pathway for Neck Lumps Referred as Urgent Suspected Head and Neck Cancer. Laryngoscope Investig. Otolaryngol..

[8] Alabi R.O., Guntinas-Lichius O., Elmusrati M., Almangush A., Tiblom Ehrsson Y., Laurell G., Mäkitie A.A. (2025). Machine learning for survival outcome in head and neck squamous cell carcinoma: A multicenter validation study. Sci. Rep..

[9] D’Cruz A.K., Vaish R., Kapre N., Dandekar M., Gupta S., Hawaldar R., Agarwal J.P., Pantvaidya G., Chaukar D., Deshmukh A. (2015). Elective versus Therapeutic Neck Dissection in Node-Negative Oral Cancer. N. Engl. J. Med..

[10] Ang K.K., Harris J., Wheeler R., Weber R., Rosenthal D.I., Nguyen-Tân P.F., Westra W.H., Chung C.H., Jordan R.C., Lu C. (2010). Human papillomavirus and survival of patients with oropharyngeal cancer. N. Engl. J. Med..

[11] Wunschel M., Neumeier M., Utpatel K., Reichert T.E., Ettl T., Spanier G. (2021). Staging more important than grading? Evaluation of malignancy grading, depth of invasion, and resection margins in oral squamous cell carcinoma. Clin. Oral Investig..

[12] Leitlinienprogramm Onkologie (2024). S3-Leitlinie Diagnostik, Therapie, Prävention und Nachsorge des Oro- und Hypopharynxkarzinoms. https://register.awmf.org/assets/guidelines/017-082OLl_S3_Diagnostik-Therapie-Praevention-Nachsorge-Oro-und-Hypopharynxkarzinom_2024-03.pdf.

[13] Pynnonen M.A., Gillespie M.B., Roman B., Rosenfeld R.M., Tunkel D.E., Bontempo L., Brook I., Chick D.A., Colandrea M., Finestone S.A. (2017). Clinical Practice Guideline: Evaluation of the Neck Mass in Adults. Otolaryngol. Head Neck Surg..

[14] Homer J.J., Winter S.C., Abbey E.C., Aga H., Agrawal R., Ap Dafydd D., Arunjit T., Axon P., Aynsley E., Bagwan I.N. (2024). Head and Neck Cancer: United Kingdom National Multidisciplinary Guidelines, Sixth Edition. J. Laryngol. Otol..

[15] NHS England (2022). NHS Cancer Programme: Faster Diagnosis Framework.

[16] Lotfallah A., Singh K., Beese R., Butler J., Gupta K.K., Davidson N., Deutsch P.G., Daultrey C., Ayshford C. (2026). Five-year outcomes of a successfully implemented One Stop Neck Lump Clinic for head and neck cancer assessment. J. Laryngol. Otol..

[17] Wang S., Hossack J.A., Klibanov A.L. (2020). From Anatomy to Functional and Molecular Biomarker Imaging and Therapy: Ultrasound Is Safe, Ultrafast, Portable, and Inexpensive. Investig. Radiol..

[18] Aulino J.M., Kirsch C.F.E., Burns J., Busse P.M., Chakraborty S., Choudhri A.F., Conley D.B., Jones C.U., Lee R.K., Expert Panel on Neurologic Imaging (2019). ACR Appropriateness Criteria® Neck Mass-Adenopathy. J. Am. Coll. Radiol..

[19] Angerer F., Zenk J., Iro H., Bozzato A. (2013). Use of a portable ultrasound system in the perisurgical assessment of head and neck patients. HNO.

[20] Alsibani A., Alqahtani A., Almohammadi R., Islam T., Alessa M., Aldhahri S.F., Al-Qahtani K.H. (2024). Comparing the Efficacy of CT, MRI, PET-CT, and US in the Detection of Cervical Lymph Node Metastases in Head and Neck Squamous Cell Carcinoma with Clinically Negative Neck Lymph Node: A Systematic Review and Meta-Analysis. J. Clin. Med..

[21] Liao L.J., Lo W.C., Hsu W.L., Wang C.T., Lai M.S. (2012). Detection of cervical lymph node metastasis in head and neck cancer patients with clinically N0 neck—A meta-analysis comparing different imaging modalities. BMC Cancer.

[22] de Bondt R.B., Nelemans P.J., Hofman P.A., Casselman J.W., Kremer B., van Engelshoven J.M., Beets-Tan R.G. (2007). Detection of lymph node metastases in head and neck cancer: A meta-analysis comparing US, USgFNAC, CT and MR imaging. Eur. J. Radiol..

[23] Sidhu P.S. (2015). Multiparametric Ultrasound (MPUS) Imaging: Terminology Describing the Many Aspects of Ultrasonography. Ultraschall Med..

[24] Huang S., Zhao Y., Jiang X., Lin N., Zhang M., Wang H., Zheng A., Ma X. (2021). Clinical Utility of Contrast-enhanced Ultrasound for the Diagnosis of Lymphadenopathy. Ultrasound Med. Biol..

[25] Gao Y., Zhao Y., Choi S., Chaurasia A., Ding H., Haroon A., Wan S., Adeleke S. (2022). Evaluating Different Quantitative Shear Wave Parameters of Ultrasound Elastography in the Diagnosis of Lymph Node Malignancies: A Systematic Review and Meta-Analysis. Cancers.

[26] Gurun E., Basaran M., Ozturk M., Cakir I.M., Gundogdu H., Duman G.N., Genc G., Akdulum I. (2026). Relationship Between Thyroid Gland Stiffness and Coronary Artery Calcium Score: A Shear Wave Elastography Study. Bratisl. Med. J..

[27] Gurun E., Ozturk M., Basaran M., Okutan A.E. (2026). Shear Wave Elastography in Musculoskeletal Imaging: A Narrative Review. J. Clin. Med..

[28] Suh C.H., Choi Y.J., Baek J.H., Lee J.H. (2017). The diagnostic performance of shear wave elastography for malignant cervical lymph nodes: A systematic review and meta-analysis. Eur. Radiol..

[29] Spiesecke P., Neumann K., Wakonig K., Lerchbaumer M.H. (2022). Contrast-enhanced ultrasound (CEUS) in characterization of inconclusive cervical lymph nodes: A meta-analysis and systematic review. Sci. Rep..

[30] Ying M., Bhatia K.S., Lee Y.P., Yuen H.Y., Ahuja A.T. (2014). Review of ultrasonography of malignant neck nodes: Greyscale, Doppler, contrast enhancement and elastography. Cancer Imaging.

[31] Möller K., Jenssen C., Lerchbaumer M.H., Hollerweger A., Yadav M., Dighe M., Serra C., Boccatonda A., Faiss S., Dietrich C.F. (2026). Lymph Node Assessment with Multiparametric Ultrasound: Normal Values, Morphologic Patterns, and Diagnostic Algorithms. Cancers.

[32] Wakonig K.M., Dommerich S., Fischer T., Arens P., Hamm B., Olze H., Lerchbaumer M.H. (2023). The Diagnostic Performance of Multiparametric Ultrasound in the Qualitative Assessment of Inconclusive Cervical Lymph Nodes. Cancers.

[33] Lerchbaumer M.H., Wakonig K.M., Arens P., Dommerich S., Fischer T. (2022). Quantitative Multiparametric Ultrasound (mpUS) in the Assessment of Inconclusive Cervical Lymph Nodes. Cancers.

[34] Bossuyt P.M., Reitsma J.B., Bruns D.E., Gatsonis C.A., Glasziou P.P., Irwig L., Lijmer J.G., Moher D., Rennie D., de Vet H.C. (2015). STARD 2015: An updated list of essential items for reporting diagnostic accuracy studies. BMJ.

[35] Ferraioli G., Barr R.G., Farrokh A., Radzina M., Cui X.W., Dong Y., Rocher L., Cantisani V., Polito E., D’Onofrio M. (2022). How to perform shear wave elastography. Part I. Med. Ultrason..

[36] Chammas M.C., Macedo T.A., Lo V.W., Gomes A.C., Juliano A., Cerri G.G. (2016). Predicting malignant neck lymphadenopathy using color duplex sonography based on multivariate analysis. J. Clin. Ultrasound.

[37] Rohan K., Ramesh A., Sureshkumar S., Vijayakumar C., Abdulbasith K.M., Krishnaraj B. (2020). Evaluation of B-Mode and Color Doppler Ultrasound in the Diagnosis of Malignant Cervical Lymphadenopathy. Cureus.

[38] Prativadi R., Dahiya N., Kamaya A., Bhatt S. (2017). Chapter 5 Ultrasound Characteristics of Benign vs Malignant Cervical Lymph Nodes. Semin. Ultrasound CT MR.

[39] Deutsche Krebsgesellschaft Ultraschallkriterien zur Differenzierung Zwischen Benignen und Malignen Zervikalen Lymphknoten bei Mundhöhlenkarzinom. https://www.krebsgesellschaft.de/unsere-themen/zertifizierung/erhebungsboegen-und-dokumente?file=files%2Fcontent%2Funsere-themen%2Fzertifizierung%2Ferhebungsboegen-und-dokumente%2Fkopf-hals-tumor-zentren%2Faktuell%2Fultraschallkriterien-differenzierung-benigne-und-maligne-zervikale-lk-bei-mhk.pdf.

[40] Eisenhauer E.A., Therasse P., Bogaerts J., Schwartz L.H., Sargent D., Ford R., Dancey J., Arbuck S., Gwyther S., Mooney M. (2009). New response evaluation criteria in solid tumours: Revised RECIST guideline (version 1.1). Eur. J. Cancer.

[41] Na D.G., Lim H.K., Byun H.S., Kim H.D., Ko Y.H., Baek J.H. (1997). Differential diagnosis of cervical lymphadenopathy: Usefulness of color Doppler sonography. AJR Am. J. Roentgenol..

[42] Bhatia K.S., Cho C.C., Tong C.S., Yuen E.H., Ahuja A.T. (2012). Shear wave elasticity imaging of cervical lymph nodes. Ultrasound Med. Biol..

[43] Chen X., Ma J., Fu Y., Mei F., Tang R., Xue H., Lin Y., Wang S., Cui L. (2024). Differential diagnosis of cervical lymphadenopathy: Integration of postvascular phase of contrast-enhanced ultrasound and predictive nomogram model. Eur. J. Surg. Oncol..

[44] Newcombe R.G. (1998). Improved confidence intervals for the difference between binomial proportions based on paired data. Stat. Med..

[45] Cai D., Wu S. (2022). Efficacy of logistic regression model based on multiparametric ultrasound in assessment of cervical lymphadenopathy—A retrospective study. Dentomaxillofac. Radiol..

[46] Yang J.R., Song Y., Jia Y.L., Ruan L.T. (2021). Application of multimodal ultrasonography for differentiating benign and malignant cervical lymphadenopathy. Jpn. J. Radiol..

[47] Takamura M., Nikkuni Y., Hayashi T., Katsura K., Nishiyama H., Yamazaki M., Maruyama S., Tanuma J.I. (2023). Comparing the Diagnostic Accuracy of Ultrasonography, CT, MRI, and PET/CT in Cervical Lymph Node Metastasis of Oral Squamous Cell Carcinoma. Biomedicines.

[48] Sumi M., Ohki M., Nakamura T. (2001). Comparison of sonography and CT for differentiating benign from malignant cervical lymph nodes in patients with squamous cell carcinoma of the head and neck. AJR Am. J. Roentgenol..

[49] Zhang J., Hao X., Yang Y., Yan C.S., Ma C., Xiao M., Gu L.S., Wang Y. (2017). Evaluation of supplementary diagnostic value of contrast-enhanced ultrasound for lymph node puncture biopsy. J. Thorac. Dis..

[50] Sun Y., Wang W., Mi C., Zhang Q., Zhang K. (2022). Differential Diagnosis Value of Shear-Wave Elastography for Superficial Enlarged Lymph Nodes. Front. Oncol..

[51] Warm J.J., Melchiors J., Kristensen T.T., Aabenhus K., Charabi B.W., Eberhard K., Konge L., von Buchwald C., Todsen T. (2024). Head and neck ultrasound training improves the diagnostic performance of otolaryngology residents. Laryngoscope Investig. Otolaryngol..

[52] Kwok M.M.K., Choong K.W.K., Virk J., Magarey M.J.R., Flatman S. (2021). Surgeon-performed ultrasound in a head and neck surgical oncology clinic: Saving time and improving patient care. Eur. Arch. Otorhinolaryngol..

[53] Künzel J., Strieth S., Wirth G., Bozzato A. (2018). Ultrasound in the Re-Staging of Cervical Metastases after Chemoradiotherapy for Head and Neck Cancer. Ultraschall Med..

[54] National Comprehensive Cancer Network (2026). NCCN Clinical Practice Guidelines in Oncology (NCCN Guidelines®): Head and Neck Cancers.

[55] Qu K., Stalker M., Hwang W.T., Cohen R.B., Sun L. (2026). Recurrent/metastatic relapse after definitive treatment for HNSCC: Timing, patterns, and survival. Front. Oncol..

[56] Haring C.T., Kana L.A., Dermody S.M., Brummel C., McHugh J.B., Casper K.A., Chinn S.B., Malloy K.M., Mierzwa M., Prince M.E.P. (2023). Patterns of recurrence in head and neck squamous cell carcinoma to inform personalized surveillance protocols. Cancer.

[57] Künzel J., Brandenstein M., Zeman F., Symeou L., Platz Batista da Silva N., Jung E.M. (2022). Multiparametric Ultrasound of Cervical Lymph Node Metastases in Head and Neck Cancer for Planning Non-Surgical Therapy. Diagnostics.

[58] Tong J., Lin T., Wen B., Chen P., Wang Y., Yu Y., Chen M., Yang G. (2023). The value of multimodal ultrasound in diagnosis of cervical lymphadenopathy: Can real-time elastography help identify benign and malignant lymph nodes?. Front. Oncol..

[59] Wüstner M., Radzina M., Calliada F., Cantisani V., Havre R.F., Jenderka K.V., Kabaalioğlu A., Kocian M., Kollmann C., Künzel J. (2022). Professional Standards in Medical Ultrasound—EFSUMB Position Paper (Long Version)—General Aspects. Ultraschall Med..

[60] Lysack J.T., Hoy M., Hudon M.E., Nakoneshny S.C., Chandarana S.P., Matthews T.W., Dort J.C. (2013). Impact of neuroradiologist second opinion on staging and management of head and neck cancer. J. Otolaryngol. Head Neck Surg..

[61] American Academy of Otolaryngology–Head and Neck Surgery Position Statement: Surgeon Performed Neck Ultrasound. https://www.entnet.org/resource/position-statement-surgeon-performed-neck-ultrasound/.

